# The mRNA-lncRNA landscape of multiple tissues uncovers key regulators and molecular pathways that underlie heterosis for feed intake and efficiency in laying chickens

**DOI:** 10.1186/s12711-023-00834-x

**Published:** 2023-10-06

**Authors:** Jingwei Yuan, Jinmeng Zhao, Yanyan Sun, Yuanmei Wang, Yunlei Li, Aixin Ni, Yunhe Zong, Hui Ma, Panlin Wang, Lei Shi, Jilan Chen

**Affiliations:** Key Laboratory of Animal (Poultry) Genetics Breeding and Reproduction, Ministry of Agriculture and Rural Affairs, Institute of Animal Science, Chinese Academy of Agricultural Sciences, Beijing, 100193 China

## Abstract

**Background:**

Heterosis is routinely exploited to improve animal performance. However, heterosis and its underlying molecular mechanism for feed intake and efficiency have been rarely explored in chickens. Feed efficiency continues to be an important breeding goal trait since feed accounts for 60 to 70% of the total production costs in poultry. Here, we profiled the mRNA-lncRNA landscape of 96 samples of the hypothalamus, liver and duodenum mucosa from White Leghorn (WL), Beijing-You chicken (YY), and their reciprocal crosses (WY and YW) to elucidate the regulatory mechanisms of heterosis.

**Results:**

We observed negative heterosis for both feed intake and residual feed intake (RFI) in YW during the laying period from 43 to 46 weeks of age. Analysis of the global expression pattern showed that non-additivity was a major component of the inheritance of gene expression in the three tissues for YW but not for WY. The YW-specific non-additively expressed genes (YWG) and lncRNA (YWL) dominated the total number of non-additively expressed genes and lncRNA in the hypothalamus and duodenum mucosa. Enrichment analysis of YWG showed that mitochondria components and oxidation phosphorylation (OXPHOS) pathways were shared among the three tissues. The OXPHOS pathway was enriched by target genes for YWL with non-additive inheritance of expression in the liver and duodenum mucosa. Weighted gene co-expression network analysis revealed divergent co-expression modules associated with feed intake and RFI in the three tissues from WL, YW, and YY. Among the negatively related modules, the OXPHOS pathway was enriched by hub genes in the three tissues, which supports the critical role of oxidative phosphorylation. Furthermore, protein quantification of ATP5I was highly consistent with *ATP5I* expression in the liver, which suggests that, in crossbred YW, non-additive gene expression is down-regulated and decreases ATP production through oxidative phosphorylation, resulting in negative heterosis for feed intake and efficiency.

**Conclusions:**

Our results demonstrate that non-additively expressed genes and lncRNA involved in oxidative phosphorylation in the hypothalamus, liver, and duodenum mucosa are key regulators of the negative heterosis for feed intake and RFI in layer chickens. These findings should facilitate the rational choice of suitable parents for producing crossbred chickens.

**Supplementary Information:**

The online version contains supplementary material available at 10.1186/s12711-023-00834-x.

## Background

Feed consumption accounts for 60 to 70% of the total production costs in poultry production. Improving feed efficiency is vital to reduce production costs and achieve sustainability for the poultry industry. Residual feed intake (RFI), which is defined as the difference between observed feed intake and feed intake predicted from metabolic body weight, body weight gain, and egg mass in laying hens, is a desirable measurement to characterize feed efficiency in livestock. Phenotypically, individuals with a lower RFI have a reduced feed intake and an improved feed efficiency with no effect on performance. Previous studies have indicated that feed intake and RFI are moderately to highly heritable [1] and are highly polygenic complex traits in laying hens [2]. It has been suggested that variation in RFI may be due to the difference in physiological processes of an animal, including feeding behavior, energy homeostasis, and nutrients absorption and metabolism processes that occur mainly in the central nervous system, metabolic organs, and digestive and absorption systems [3]. Many studies have been carried out separately on different feed efficiency-related tissues, such as the hypothalamus, liver, adipose tissue, muscle, and duodenum mucosa to elucidate the molecular mechanisms that regulate feed efficiency in chickens [4]. In growing pigs, the immune response, defense against pathogens, and oxidative stress mechanisms that are shared among the liver, muscle, and adipose tissues were found to regulate differences in feed efficiency between individuals [5]. Sun et al. [6] characterized 19 common and tissue-specific gene markers that were associated with feed efficiency using multi-tissue transcriptome analysis. These findings suggested that gene expression that regulates common and unique functions across multi-tissues drives phenotypic variation in feed efficiency. However, how the shared and interactive pathways among tissues regulate feed intake and efficiency is largely unknown in laying chickens. Analysis of co-expression networks across tissues may be a promising approach to unravel the biological pathways and processes that underlie feed intake and efficiency [7].

A cross between genetically-distant parents often produces hybrids with superior growth rate, production, etc. compared to its parents. Thus, crossbreeding is widely promoted as an efficient strategy to increase farmers’ income through improvement of productivity of livestock [8]. Improvement via crossbreeding is based on the use of heterosis, which significantly increases the productivity of animals, by 15 to 50% [9]. Although heterosis is widely exploited, the underlying molecular mechanisms are poorly understood. Classically, genetic models that include dominance, overdominance, heterozygosity, epistasis, and nonallelic interactions have been used to explain the phenomenon of heterosis, but the debate on models to explain heterosis is still ongoing [10]. High-throughput omics technologies provide opportunities to study genome-wide changes in gene expression and to uncover regulatory network changes related to heterosis phenotypes, which could provide important insights into unraveling the molecular mechanism of heterosis [11–14].

Hypothalamus, liver, and duodenum are key organs that are involved in feeding behavior, energy metabolism, digestion, and total metabolism in layer chickens. Therefore, transcriptomic analysis of these organs may reveal regulatory factors and elucidate molecular mechanisms that are crucial for heterosis in feed intake and efficiency. In this study, we constructed a reciprocal crossbred chicken population and measured feed intake and efficiency during the laying period. The global mRNA and lncRNA expression patterns across the three tissues from purebred and crossbred hens were assessed using lncRNA-Seq based transcriptome analysis and linked to phenotype traits and their heterosis. The aim of our work was to provide new insights into the molecular basis of heterosis for feed intake and efficiency in chickens.

## Methods

### Experimental population

In this study, White Leghorn (WL) and Beijing You chicken (a Chinese indigenous breed, BY) were mated to produce purebred progenies and reciprocal crosses. WL was introduced from Canada and has been selected for egg production traits for more than 20 generations, whereas BY has been selected for egg production traits for 15 generations. The full mating procedure was published previously [15]. Briefly, 30 BY roosters with good semen quality were randomly mated with 150 BY hens and 150 WL hens to generate YY and YW, respectively, and 30 WL roosters with good semen quality were randomly mated with 150 different WL hens and 150 different BY hens to generate WL and WY, respectively. A 2-generation pedigree including 60 sires and 417 dams from the parental generation and 984 hens from the F1 generation was produced. Among these animals, only the F1 birds were measured for the traits of interest. All birds were hatched on the same day and housed in identical pens under standard management conditions on the experimental farm of IAS-CAAS. Each bird was raised in an individual cage and ad libitum access to water and to a commercial corn–soybean diet that met National Research Council requirements during the feeding trial were provided.

### Measurement of feed efficiency traits and heterosis

Feed intake was collected on each hen during a 4-week (43 to 46 weeks of age) trial period by providing feed in an individual container for each hen. Feed was manually added daily and the total weight was recorded to compute daily feed intake (DFC). Egg number and weight were recorded daily to calculate total egg mass. Body weight was measured at the beginning and end of the test for each hen to calculate the mean body weight (BW), metabolic BW (BW raised to the power of 0.75), and daily body weight gain (BWG). Average daily egg mass (DEM) was calculated as the total egg mass divided by the number of test days. Feed conversion ratio (FCR) was calculated as the ratio of DFC to DEM. After removing data on hens without eggs, the remaining 904 hens, including 198 WL, 245 YY, 238 YW, and 223 WY, were used to calculate residual feed intake (RFI) as the residual from a regression model of DFC on metabolic BW (MBW), BWG, and DEM [16] using the lm() function in the R software.

We then analyzed the phenotype by fitting the following animal model with pedigree information using the AI-REML algorithm [17] implemented in the DMU software (http://dmu.agrsci.dk):

1 $$\mathbf{y}=\mathbf{1}\upmu +\mathbf{X}\mathbf{b}+{\mathbf{Z}}_{\mathbf{a}}\mathbf{a}+{\mathbf{Z}}_{\mathbf{c}}\mathbf{c}+\mathbf{e},$$

where $\mathbf{y}$ is the vector of observations,$\mathbf{1}$ is a vector of ones, $\upmu$ is the overall mean, $\mathbf{b}$ is the vector of fixed effects of groups, $\mathbf{X}$ is the incidence matrix of $\mathbf{b}$, $\mathbf{a}$ and $\mathbf{c}$ are vectors of random additive genetic and random non-additive effects, respectively, of each bird ${\mathbf{Z}}_{\mathbf{a}}$ and ${\mathbf{Z}}_{\mathbf{c}}$ are the incidence matrices of the additive and non-additive effects, respectively, and $\mathbf{e}$ is the vector of residuals. Then, mid-parent heterosis ($\mathrm{H}$) was calculated as:

2 $$\mathrm{H}= \frac{\overline{\mathrm{F} }-(\overline{{\mathrm{P} }_{\mathrm{f}}}+ \overline{{\mathrm{P} }_{\mathrm{m}}})/2}{(\overline{{\mathrm{P} }_{\mathrm{f}}}+\overline{{\mathrm{P} }_{\mathrm{m}}})/2},$$

where $\overline{\mathrm{F} }$, $\overline{{\mathrm{P} }_{\mathrm{f}}}$ and $\overline{{\mathrm{P} }_{\mathrm{m}}}$ denote the average trait values estimated by the animal model for the reciprocal crosses, the paternal and maternal lines, respectively. Significance of heterosis was estimated by a transformed Student’s t test value based on the formula:

3 $$\mathrm{t}= \frac{\mathrm{H}}{2\sqrt{\frac{\sum {({\mathrm{F}}_{\mathrm{i}}-\overline{\mathrm{F} })}^{2}}{\mathrm{n}-1}}/\left[(\overline{{\mathrm{P} }_{\mathrm{f}}}+\overline{{\mathrm{P} }_{\mathrm{m}}})\times \sqrt{\mathrm{n}}\right]},$$

where ${\mathrm{F}}_{\mathrm{i}}$ is the phenotype of the $\mathrm{i}$-th bird from the reciprocal crosses and $\mathrm{n}$ is the number of WY or YW birds [14]. We obtained the *P* value using the pt() function in the R software based on the t value and the degrees of freedom. A *P* value < 0.05 was considered to be significant.

### RNA extraction and sequencing

Eight hens from each group with records that were close to each group mean for RFI were euthanized by cervical dislocation and the hypothalamus, liver, and duodenum mucous were isolated for subsequent RNA sequencing. Total RNA was extracted using the TRIzol® Reagent (Invitrogen, USA) according to the manufacturer’s instructions. The extracted RNA was first evaluated by 1% agarose gel electrophoresis. RNA purity, concentration, and integrity of all eligible RNA samples were determined using a NanoPhotometer® spectrophotometer (IMPL EN, CA, USA). Ninety-six qualified samples were used for RNA sequencing, including 31 hypothalamus, 33 liver, and 32 duodenum mucosae samples (see Additional file 1: Table S1). Approximately 3 μg of RNA per sample was subjected to RNA-seq library construction using the NEBNext® UltraTM RNA Library Prep Kit (Illumina, USA) according to the manufacturer’s guide. After PCR amplification and purification, 150-bp paired-end sequencing was performed on the Illumina nova 6000 platform (Illumina Inc., San Diego, CA, USA).

### Quality control, mapping, and transcriptome assembly

Raw reads were removed based on the following parameters: (a) those containing adaptors; (b) with more than 10% unknown nucleotides; and (c) with more than 50% low-quality bases (Qphred ≤ 20). After quality control, high-quality reads with Q20 > 95% were mapped to the chicken reference genome (GRCg6) using the Hisat2 (v2.1.0) program [18]. Then, the mapped data were used as inputs for Stringtie (v2.1.5) [19] to assemble aligned reads into transcripts [20], using default parameters (−f 0.01, −c 1). The number of mapped reads was normalized into fragments per kilobase million (FPKM). Expressed genes were defined as having a FPKM > 0 in at least one sample, and tissue-expressed genes were defined as having a FPKM > 0 in more than 30% of the samples from a particular tissue.

### Identification of lncRNA

We used strict filters to identify potential lncRNA from the 96 samples. First, the assembled transcripts were merged by Stringtie with parameters (−m 200, −F 1, −c 2) to remove transcripts shorter than 200 bp, with low expression levels (FPKM < 1), and low coverage (< 2). Second, the merged transcripts were matched to the Ensembl gene annotation file using the GffCompare (v 0.12.6) software with parameters (−M) to filter out transcripts with only one exon. Third, transcripts with a class code “i”, “u” and “x” were extracted for the further analysis. Subsequently, sequences of known lncRNA from the ALDB [21] and NONCODE databases [22] were downloaded for aligning the putative transcripts with parameters (e value ≤ 1 × 10e−6, perc_identity ≥ 90%) implemented in BLAST (version 2.2.26). We calculated the coding potential for the remaining transcripts using the CPC [23], CNCI [24], and PLEK [25] tools. All putative transcripts were translated into amino acid sequences to remove transcripts that contained known protein domains through open reading frames. The HMMER [26] software package was used to identify any known protein domain by searching against the Pfam database (Pfam 32.0). Transcripts with significant Pfam hits were excluded and those without coding potential were considered as candidate sets of lncRNA. Finally, the cis- and trans-acting relationship between lncRNA and potential protein-coding genes was predicted according to their distances (gene located within 100 kb of the lncRNA) [27] and expression correlations (correlation coefficient $\ge$ 0.95 or $\le$ −0.95 between gene expression and lncRNA expression) [28].

### lncRNA and gene inheritance patterns

The lncRNA and gene count matrix was generated using the featureCounts software (v2.0.3) [29] and the resulting count data was “regularized log” transformed using the rlog() function for principal component analysis (PCA), which was visualized by the plotPCA () function in the DESeq2 package (v.1.16.1) [30]. The confidence interval of the PCA results was 0.95, which was calculated and plotted using the stat_ellipse() function in the ggplot2 package. Differential expression analysis between the two purebred lines (WL vs. YY) and between the reciprocal crosses and purebred lines (WY vs. WL WY vs. YY, YW vs. WL and YW vs. YY) was carried out using the DESeq2 package (v.1.16.1). lncRNA and genes with an adjusted *P* value < 0.05 and |Log2Fold change|> 1.2 were considered differentially expressed in the corresponding contrast. We used the average FPKM value of the purebred and crossbred means and the average of the two purebred means to evaluate different inheritance patterns of lncRNA and genes, which were further classified into three inheritance patterns including additivity, dominance, and overdominance (see Additional file 2: Table S2) [31]. Briefly, additivity (4 and 10) occurred when the expression was significantly (adjusted *P* value < 0.05) different between the two parental lines, and the expression of the reciprocal crosses (WY or YW) was not significantly different (adjusted *P* value ≥ 0.05) from the average of the two parental line means. Expression in WY or YW that was not significantly different from that in one parental line but significantly (adjusted *P* value < 0.05) higher or lower than that in the other parental line was regarded as dominance including high parent pattern (3, 11) and low parent pattern (5, 9). Mean expression in WY or YW that was significantly (adjusted *P* value < 0.05) higher (or lower) than that in both parental lines was considered as overdominance including above high-parent pattern, above parent pattern (1, 2 and 12) or underdominance including below low-parent pattern, below parent pattern (6, 7 and 8).

### Weighted gene co-expression network analysis (WGCNA)

Co-expression modules were constructed separately for the normalized expression data of the hypothalamus, liver, and duodenum mucosa using WGCNA in R [32]. First, an appropriate “soft-thresholding” value was selected for each tissue by plotting the strength of the correlation against a series of soft threshold powers (from 1 to 30), with a signed pairwise correlation matrix generated by Pearson’s product moment correlation. The correlation matrix was subsequently transformed into an adjacency matrix, in which node and edge corresponded to lncRNA/gene and the connection strength between lncRNA/gene, respectively. Each adjacency matrix was normalized using a topological overlap function. Hierarchical clustering was performed using average linkage. The hierarchical cluster tree was cut into modules using the dynamic tree cut algorithm with a minimum module size of 50 lncRNA/gene. We merged modules if the correlation between their eigengenes (defined as the first principal component of their gene expression values) was greater or equal to 0.25. After obtaining modules for each tissue, the module eigengene that summarized as the first principal component of expression data was calculated with the “ModuleEigengenes” function. Correlation analysis between a module and the trait was performed with the “corPvalueStudent” function based on the module eigengene, and *P* < 0.01 was set for statistical significance.

We assessed the preservation of identified modules across the three tissues using the “modulePreservation” function implemented in WGCNA. The module preservation approach takes “reference” and “test” network modules as input and calculates statistics for three preservation classes: (i) density-based statistics, which assess the similarity of lncRNA/genes connectivity patterns between a reference network module and a test network module; (ii) separability-based statistics, which examine whether test network modules remain distinct in the reference network modules; and (iii) connectivity-based statistics, which are based on the similarity of connectivity patterns between lncRNA/genes in the reference and test networks [13]. The Zsummary score was used to measure preservation, with a value greater than 10 suggesting that the module is strongly preserved between the reference and test network modules, a value between 2 and 10 indicating weak to moderate preservation, and a value less than 2 indicating no preservation [33].

### Gene ontology and pathway analysis

Gene ontology (GO) enrichment analysis and Kyoto Encyclopedia of Genes and Genomes (KEGG) pathway analysis of candidate genes were performed using the ClusterProfiler package implemented in R. The false discovery rate (FDR) method was used to adjust the *P* values for multiple testing. GO terms and KEGG pathways with adjusted *P* value < 0.05 were considered to be significantly enriched.

### Western blot

To further demonstrate that non-additively expressed genes were related to negative heterosis for DFC and RFI, we performed a Western blot to quantify the protein expression of candidate genes in WL, YY, and YW. We randomly selected six birds from the purebreds and crossbreds to extract total protein from liver tissue, using a total protein extraction kit (Solarbio, Beijing, China), while protein concentration was determined using a BCA protein determination kit (TIANGEN, Beijing, China) according to the manufacturer's instructions. Equivalent amounts of total protein from each bird were separated by 15% sodium dodecyl-sulfate polyacrylamide gel electrophoresis (SDS-PAGE) and transferred to a polyvinylidene difluoride (PVDF) membrane. The membrane was blocked with QuickBlock™ blocking buffer (Beyotime, Shanghai, China) for 1 h at room temperature. Then, the membranes were incubated with specific primary antibodies at room temperature for 1.5 h in the blocking solution under agitation, including anti-ATP5I (Proteintech, Wuhan, China; diluted 1:1000). After washing, they were incubated with a specific secondary antibody (Servicebio, Chengdu, China; diluted 1:5000). Finally, enhanced chemiluminescence (ECL) luminous fluid (Beyotime) was used for detection.

## Results

### Feed efficiency and relevant traits

A multivariate linear model was fitted to estimate RFI with a goodness of fit (R^2^) of 0.38 (see Additional file 3: Table S3). For the two purebreds and their reciprocal crosses, the adjusted DFC ranged from 90.8 to 103.3 g/d, and the adjusted DEM ranged from 36.8 to 52.9 g/d. The adjusted RFI ranged from – 3.14 to 3.72 g/d and the adjusted FCR ranged from 2.06 to 3.33 (Table 1). The distribution of the phenotypic values is shown in Additional file 4: Figure S1. Based on Eqs. (2) and (3), YW crossbreds showed significant heterosis for both DFC and RFI, while WY crossbreds showed significant heterosis for RFI, FCR, and DEM. In practice, more feed intake means more cost and larger RFI means poorer feed efficiency. Therefore, the estimates of heterosis for DFC and RFI of YW were considered negative, and the estimates of heterosis for RFI of WY were considered positive.

**Table 1:** Adjusted mean values of phenotypes and their estimates of heterosis

Traits	Adjusted value (Mean ± SE)				Heterosis	
	WL	WY	YW	YY	WY	YW
RFI, g/d	1.77 ± 0.72	− 3.14 ± 0.75	3.72 ± 0.84	− 2.26 ± 0.62	1181.63%^*^	− 1618.37%^*^
DFC, g/d	100.27 ± 1.00	94.87 ± 0.98	103.28 ± 1.10	90.77 ± 0.91	− 0.68% ^ns^	8.12%^**^
FCR, g/g	2.67 ± 0.37	2.06 ± 0.04	2.94 ± 0.33	3.33 ± 0.38	− 31.33%^*^	− 2.00%^ns^
DEM, g/d	52.90 ± 0.91	47.80 ± 0.58	45.45 ± 0.69	36.77 ± 0.58	6.61%^**^	1.37%^ns^

*RFI* residual feed intake, *DFC* daily feed consumption, *FCR* feed conversion ratio, *DEM* daily egg mass, *WL* White Leghorn chicken, *YY* Beijing You chicken, *WY* and *YW* reciprocal crosses, respectively

*ns* not significant (*P* ≥ 0.05)

*SE* standard error

*P < 0.01.

### Overview of the sequencing data

**Fig. 1:**
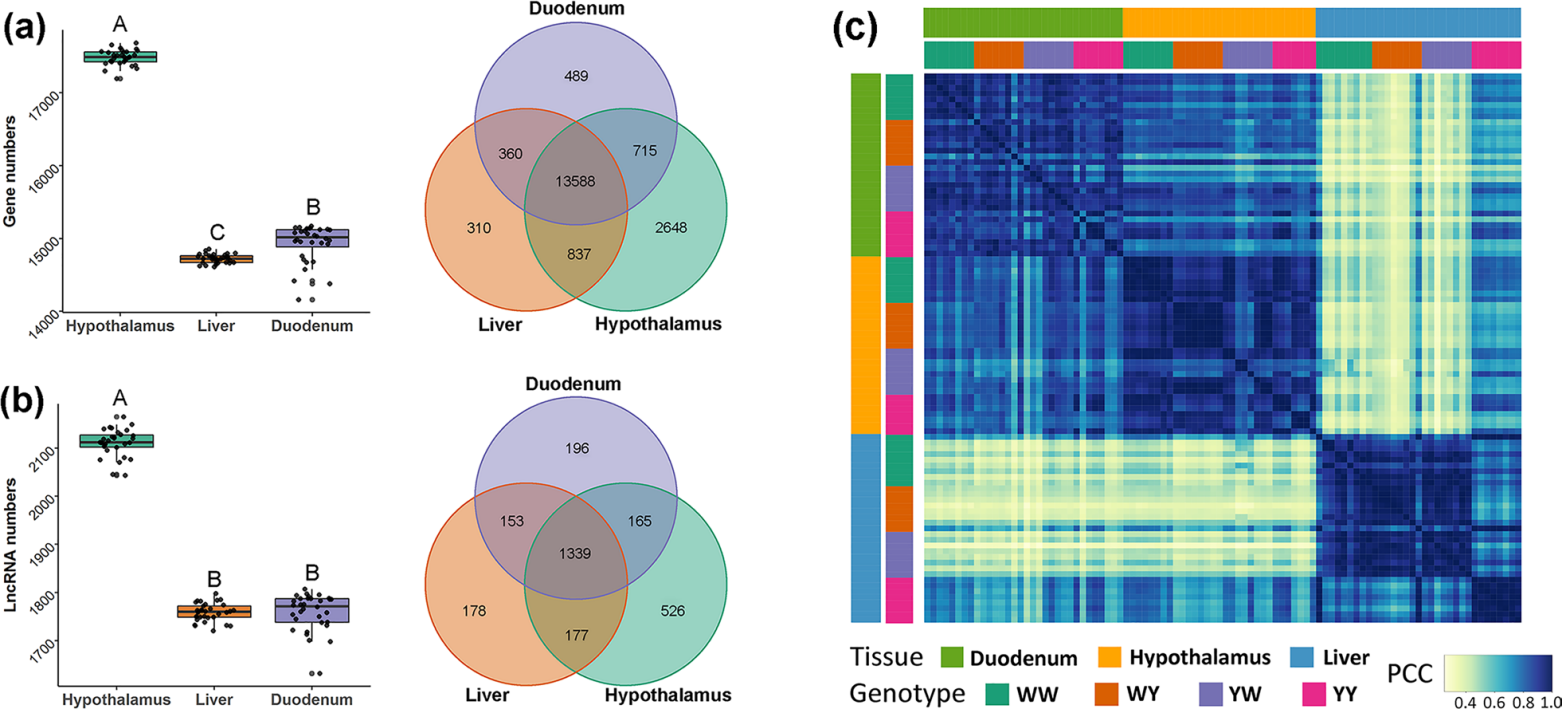
Transcriptome landscape of the hypothalamus, liver, and duodenum mucosa in White Leghorn (WL) and Beijing You chicken (YY), and their reciprocal crosses (WY and YW). **a** Average number of expressed genes, shared genes, and uniquely expressed genes in three tissues. Boxes with different letters differ significantly in the number of expressed genes. The Venn figure was plotted with all the expressed genes in each tissue. **b** Average number of expressed lncRNA, shared lncRNA, and uniquely expressed lncRNA in the three tissues. Boxes with different letters differ significantly in the number of expressed lncRNA. The Venn figure was plotted with all expressed lncRNA in each tissue. **c** Pairwise Pearson correlation coefficients (PCC) between each pair of global gene expression

In total, 3,746,515,304 clean reads and 1,112,164,813,014 bp were generated from RNA sequencing of the 96 samples, among which an average of 39.33, 40.74, and 36.96 million clean reads were obtained for, respectively, the hypothalamus, liver, and duodenum mucosa. Among these, 94.63, 95.86, and 93.62% of the clean reads were mapped to the chicken genome GRCg6a for, respectively, the hypothalamus, liver, and duodenum mucosa. Based on the mapped reads, we identified 23,117 genes, among which 22,620, 20,556, and 20,839 were expressed in, respectively, the hypothalamus, liver, and duodenum mucosa. Summary statistics of the sequence data are in Additional file 1: Table S1. As shown in Fig. 1, we identified significant differences in the average number of expressed genes between the hypothalamus (17,485 ± 106), liver (14,722 ± 61), and duodenum mucosa (14,932 ± 258). Overlaps of the expressed genes in the three tissues showed that 13,588 genes were shared by the three tissues, and 2648, 310, and 489 genes were uniquely expressed in the hypothalamus, liver, and duodenum mucosa, respectively (Fig. 1a). Similarly, a significantly larger average number of lncRNA was identified in the hypothalamus (2111 ± 28), while this number was comparable between the liver (1759 ± 18) and duodenum mucosa (1759 ± 38). Among the tissue-expressed lncRNA, 1339 lncRNA were expressed in all three tissues, and 526, 178, and 196 lncRNAs were uniquely expressed in the hypothalamus, liver and duodenum mucosa, respectively (Fig. 1b). Moreover, the global gene expression profiles within a given tissue were closely correlated between the purebreds and crossbreds, indicating highly conserved transcriptional features in the purebreds and reciprocal crossbred lines, which is the basis for identifying the core gene regulatory network that are associated with the significantly heterosis of the crossbreds compared with its parents. The gene expression profiles for hypothalamus and duodenum mucosa were highly correlated, while the expression profile for liver was moderately correlated with that for the other two tissues (Fig. 1c). These correlations are consistent with the high proportion of shared genes among the three tissues.

### Divergent inheritance of genes and lncRNA leads to different levels of heterosis for feed intake and efficiency

**Fig. 2:**
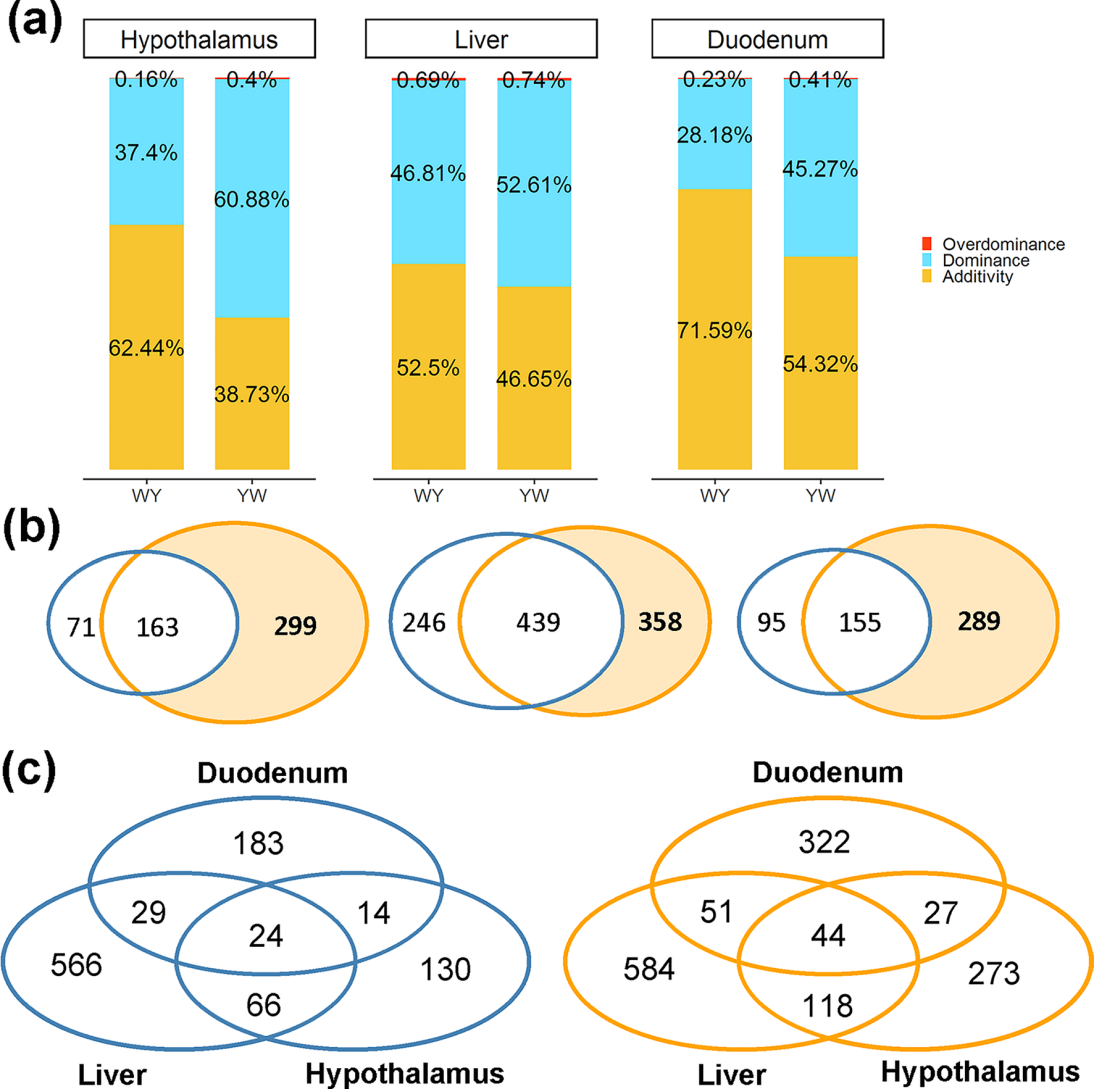
Mode of inheritance of gene expression in the hypothalamus, liver, and duodenum mucosa in the reciprocal crosses (WY and YW). **a** The proportion of additive, dominant, and over-dominant genes in the three tissues. **b** The Venn plot for non-additive genes in WY (blue circle) and YW (orange circle). From left to right, the plot represents the hypothalamus, liver, and duodenum mucosa, respectively. **c** The shared and unique non-additively expressed genes in the three tissues for WY (blue circle) and YW (orange circle)

Principal component analyses (PCA) were performed to compare the expression of the genes between purebreds and crossbreds (see Additional file 4: Figure S2a) and of lncRNA (see Additional file 4: Figure S2b), separately in the hypothalamus, liver, and duodenum mucosa. The results show that, for the three tissues, the two purebreds were separated from each other, while the two crossbreds were clustered in between the purebreds and closer to the WL chickens. We identified 234, 685, and 250 non-additive genes for WY in the hypothalamus, liver, and duodenum mucosa, respectively, and 462, 797, and 444 non-additive genes for YW in the hypothalamus, liver, and duodenum mucosa, respectively. The detailed numbers of genes for each expression pattern are provided in Additional file 5: Table S4. For WY, additive genes were predominant across the three tissues, while dominance was the principal gene inheritance pattern for YW and accounted for 60.9, 52.6, and 45.3% of the total number of non-additive genes in the hypothalamus, liver, and duodenum mucosa, respectively. Genes with overdominance were rare across all tissues in both crossbreds, accounting for 0.16 to 0.74% of all genes (Fig. 2a). Given that the estimates of heterosis for feed intake and RFI were significantly different between WY and YW, we detected both unique and common non-additive genes between WY and YW. As shown in Fig. 2b, the number of YW-specific non-additive genes (YWG) was larger than the number of such genes for WY (WYG) across the three tissues, and the YWG were predominant in the hypothalamus and duodenum mucosa. Moreover, tissue-specific genes accounted for 55.6 to 82.6% and for 59.1 to 73.3% of the total number of non-additive genes for each tissue in WY (130 for the hypothalamus, 566 for the liver, and 183 for the duodenum mucosa) and YW (273 in the hypothalamus, 584 in the liver, and 322 in the duodenum mucosa), respectively (Fig. 2c). Regarding lncRNA, the proportion of additive lncRNA was greater than 50% of the total lncRNA, and thus represents the major inheritance pattern for the three tissues for the two crossbreds (see Additional file 4: Figure S3a). For WY, we identified 56, 186, and 62 non-additively expressed lncRNA for the hypothalamus, liver, and duodenum mucosa, respectively. Similarly, for YW, 77, 177, and 88 non-additive lncRNA were identified for the hypothalamus, liver, and duodenum mucosa, respectively (see Additional file 5: Table S4). Similar to the gene expression pattern, the YW-specific non-additive lncRNA (YWL) were predominant in the hypothalamus and duodenum mucosa, accounting for 61.04 and 62.07% of all genes, respectively (see Additional file 4: Figure S3b).

### Functional annotation of non-additive genes and lncRNA

**Fig. 3:**
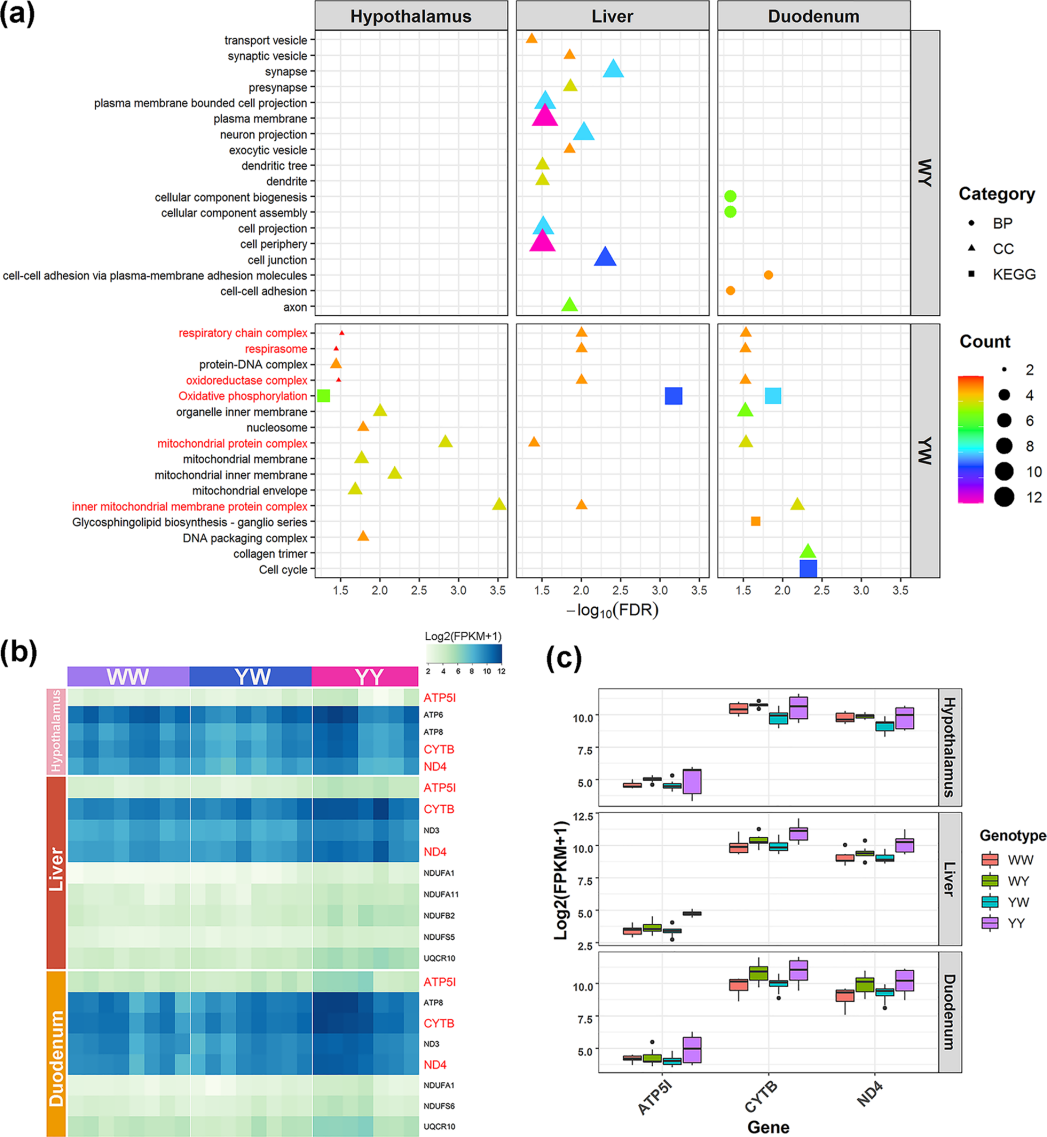
Functional annotation of non-additively expressed genes that are related with heterosis for feed intake and efficiency in the hypothalamus, liver, and duodenum mucosa. **a** Significant GO terms and KEGG pathways of non-additive genes in the reciprocal crosses. Descriptions in red represent the GO terms and KEGG pathway shared by the three tissues. **b** Expression profile of non-additive genes involved in the oxidative phosphorylation pathway for White Leghorn (WL) and Beijing You chicken (YY), and their reciprocal cross (YW). Symbols in red represent the shared genes in the three tissues. **c** Relative expression of the shared genes in the three tissues for WL and YY, and their reciprocal crosses (YW and WY). The shared genes in the three tissues are non-additively expressed in YW and additively expressed in WY, respectively

The divergent expression patterns of the genes between the two crossbreds in the hypothalamus, liver, and duodenum mucosa are putatively associated with differences in levels of heterosis for feed intake and RFI. Thus, we tested for enrichment of crossbred-specific non-additive genes against GO and KEGG pathways databases to detect biological process (BP), cellular components (CC), molecular functions (MF), and pathways associated with DFC and RFI for the three tissues (Fig. 3a). For WY, the crossbred-specific non-additive genes were significantly enriched for several CC, including for cell projection and periphery, synapse, and axon in the liver, and for various BP, such as cellular component biogenesis, nitrogen metabolism, and cell–cell adhesion in the duodenum mucosa. No GO terms and KEGG pathways were identified to be enriched in the hypothalamus. For YW, unique non-additive genes were significantly enriched for different GO terms and KEGG pathways for the three tissues (Fig. 3a). Notably, the oxidative phosphorylation (OXPHOS) pathway and mitochondrial components were enriched for all three tissues (Fig. 3a), including the mitochondrial protein complex, the inner mitochondrial membrane protein complex, oxidoreductase complex, and the respiratory chain complex and respirasome. The mitochondrial components genes were all involved in the OXPHOS pathway (see Additional file 6: Table S5).

The identical KEGG pathway and cellular components that were shared among the three tissues from YW suggest that oxidative phosphorylation and mitochondrial components have a critical role in the negative heterosis for feed intake and efficiency. For YW, the number of non-additive genes that were associated with the enriched OXPHOS pathway was equal to 5, 9, and 8 in the hypothalamus, liver, and duodenum mucosa, respectively. These genes were related to the ubiquinone oxidoreductase subunit, cytochrome b, ATP, and ubiquinol-cytochrome c reductase. Among these, *ATP5I*, *CYTB,* and *ND4* were common to the three tissues (Fig. 3b). These three genes showed additive patterns in WY and low-parent non-additive patterns in YW, respectively, with the expression level being significantly higher in YY than in WL, and YW and WL having similar expression levels (Fig. 3c).

**Fig. 4:**
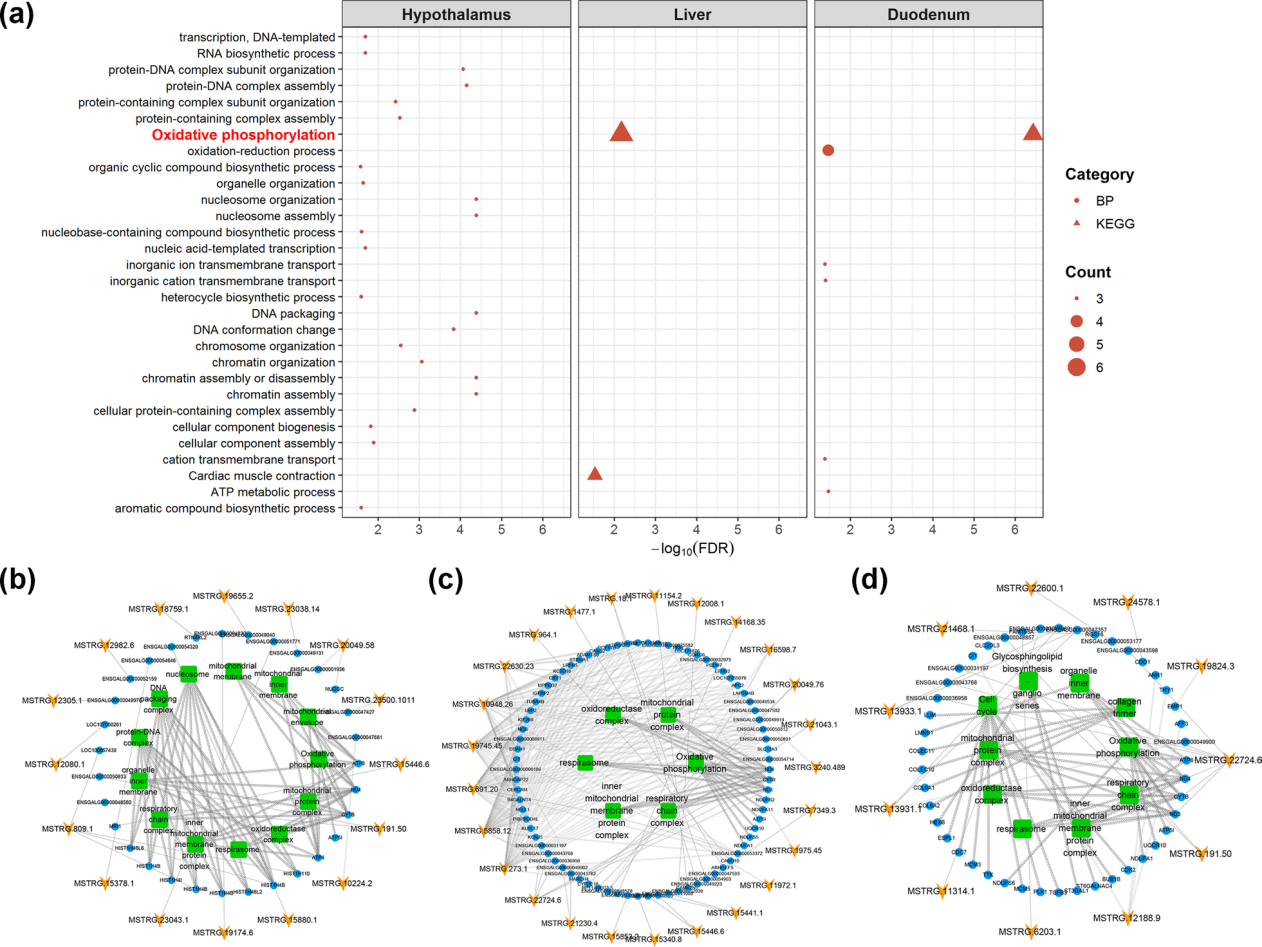
Non-additively expressed lncRNA related to negative heterosis for feed intake and efficiency in the hypothalamus, liver, and duodenum mucosa in YW. **a** Significant GO terms and KEGG pathways of non-additively expressed genes targeted by YW-specific non-additively expressed lncRNA in the three tissues. Description in red represents the KEGG pathway shared by the liver and duodenum mucosa. The interaction network among the non-additively expressed lncRNA, targeted non-additively expressed genes and significantly GO terms/KEGG pathways in the hypothalamus (**b**), liver (**c**), and duodenum mucosa (**d**), respectively, for YW. The yellow arrows, blue circles, and green squares represent lncRNA, genes and GO terms/pathways, respectively

To understand the possible biological function of the lncRNA in the negative heterosis for feed intake and RFI, we investigated the expression patterns of the unique non-additive lncRNA and their co-expressed non-additive gene targets in the three tissues of YW. First, we identified 4396 lncRNA that were significantly correlated with 13,697 genes in the 96 samples, including 56,441 cis-acting pairs (see Additional file 7: Table S6) and 138,593 trans-acting pairs (see Additional file 8: Table S7). Based on the cis- and trans-acting relationships between genes and lncRNA, we identified 36, 472, and 39 gene-lncRNA pairs in YW for which both gene and lncRNA were non-additively expressed in the hypothalamus, liver, and duodenum mucosa, respectively (see Additional file 9: Table S8 and Additional file 10: Table S9). The GO and KEGG pathway analyses revealed that the OXPHOS pathway was significantly enriched among non-additive genes that were targeted by YWL in both the liver and duodenum mucosa (Fig. 4a), which suggests that lncRNA contribute to the expression of negative heterosis for feed intake and efficiency by regulating the expression of oxidative phosphorylation genes. We further extracted 16, 25, and 11 non-additive lncRNA that were related to 33, 78, and 33 non-additive genes that were associated with significantly enriched GO terms and KEGG pathways for hypothalamus, liver, and duodenum mucosa, respectively, in YW. Among these lncRNA, *MSTRG.22724.6* cis-targeted the *CYTB*, *ND3,* and *ND4* genes that participate in the oxidative phosphorylation pathway in the liver and duodenum mucosa (Fig. 4b–d).

### Co-expression networks related to heterosis of feed intake and efficiency

**Fig. 5:**
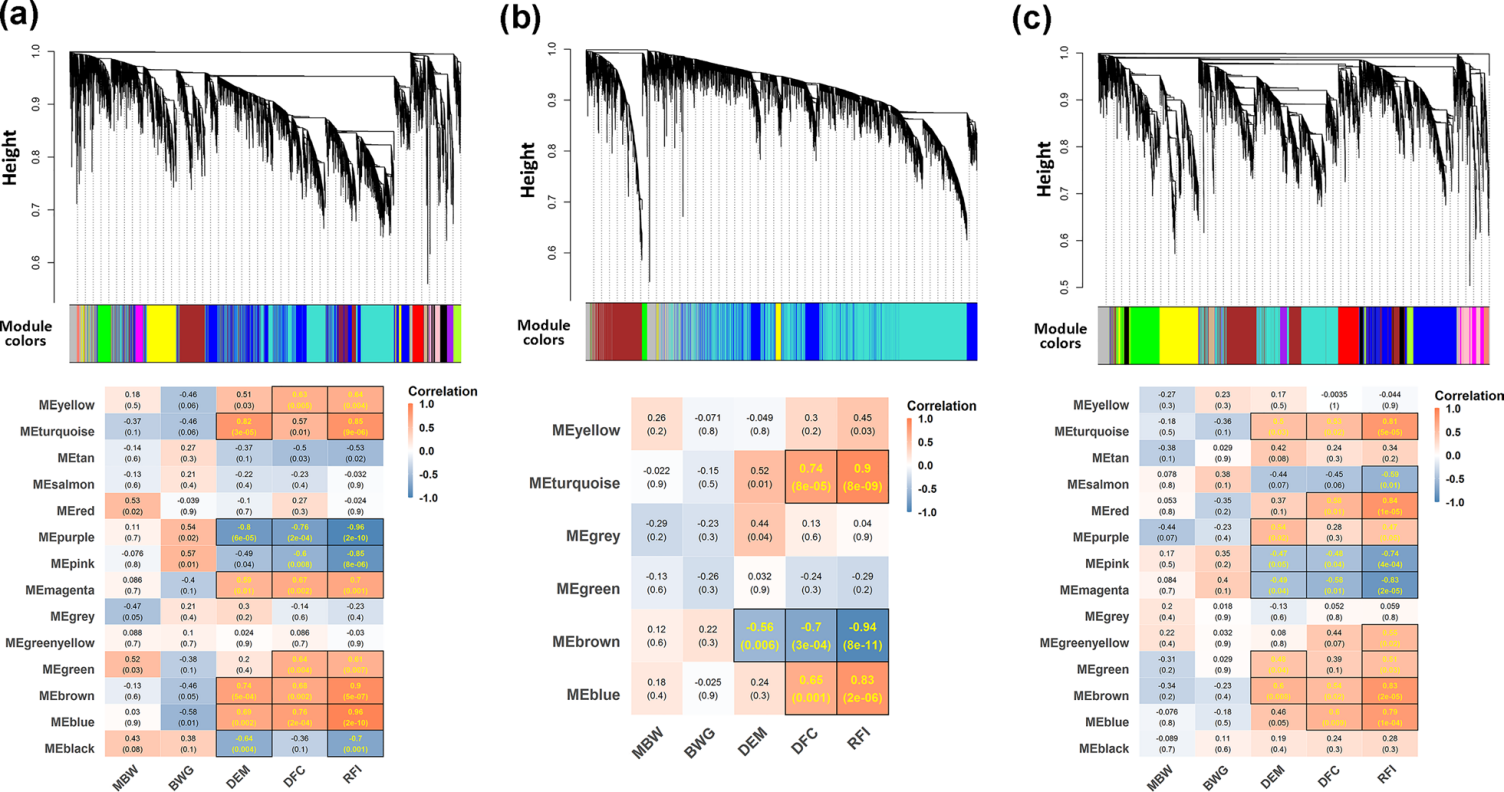
Weighted gene co-expression network analysis for the YW reciprocal cross and its parents (WL and YY). The hierarchical cluster dendrogram and module-trait relationships were plotted for the hypothalamus (**a**), liver (**b**), and duodenum mucosa (**c**), respectively. Heatmap colors indicate positive/negative Pearson correlation coefficients. Correlation coefficients and P-values are shown within the cells (yellow font, P < 0.05). *MBW* metabolic body weight, *BWG* body weight gain, *DEM* daily egg mass, *DFC* daily feed intake and *RFI* residual feed intake

Feed intake and RFI are assumed to be regulated by a transcriptional regulatory network involving multiple genes and lncRNA. Thus, we took advantage of our high-resolution multi-tissue transcriptome atlases to gain insight into heterosis for feed intake and efficiency in the crossbred YW. Based on WGCNA, we constructed the mRNA-lncRNA co-expression networks for each tissue in WL, YY, and YW. In WGCNA, the top 40% most variable genes and lncRNA were used to construct a signed, scale-free network for hypothalamus (n = 9382), liver (n = 7846), and duodenum mucosa (n = 8046), according to their topological overlap matrix of expression correlations generated by introducing the corresponding power of 18, 16 and 18 (see Additional file 4: Figure S4). As shown in Fig. 5, 14, 6, and 14 gene-lncRNA co-expression modules were identified from the hypothalamus, liver and duodenum mucosa, respectively, as identified by the different colors. Correlations between modules and feed efficiency-related traits were calculated and are shown on the module-trait relationship heatmap. We identified 22 gene modules that had significant correlations with DFC or RFI across the three tissues, but no modules that were significantly correlated with BWG or MBW. Among the 22 modules that were significantly correlated with DFC or RFI, MEblue, MEbrown, MEgreen, MEmagenta, and MEyellow were significantly positively correlated with both DFC (0.63–0.76, P < 0.01) and RFI (0.61–0.96, *P* < 0.01), while MEpink and MEpurple were significantly negatively correlated with both traits in the hypothalamus (−0.96 to -0.60,* P* < 0.01) (Fig. 5a). For liver, MEblue and MEturquoise were positively correlated with both DFC (0.65 and 0. 74,* P* < 0.01) and RFI (0.83 and 0.90,* P* < 0.01), and MEbrown was negatively correlated with both traits (− 0.7 and − 0.94,* P* < 0.01) (Fig. 5b). For duodenum mucosa, four modules were positively correlated with both DFC (0.53–0.60,* P* < 0.05) and RFI (0.79–0.84, P < 0.01), including MEblue, MEbrown, MEred and MEturquoise, while the MEmagenta and MEpink modules were negatively correlated with both DFC (− 0.58 to − 0.48,* P* < 0.05) and RFI (− 0.83 to − 0.74,* P* < 0.01) (Fig. 5c).

**Fig. 6:**
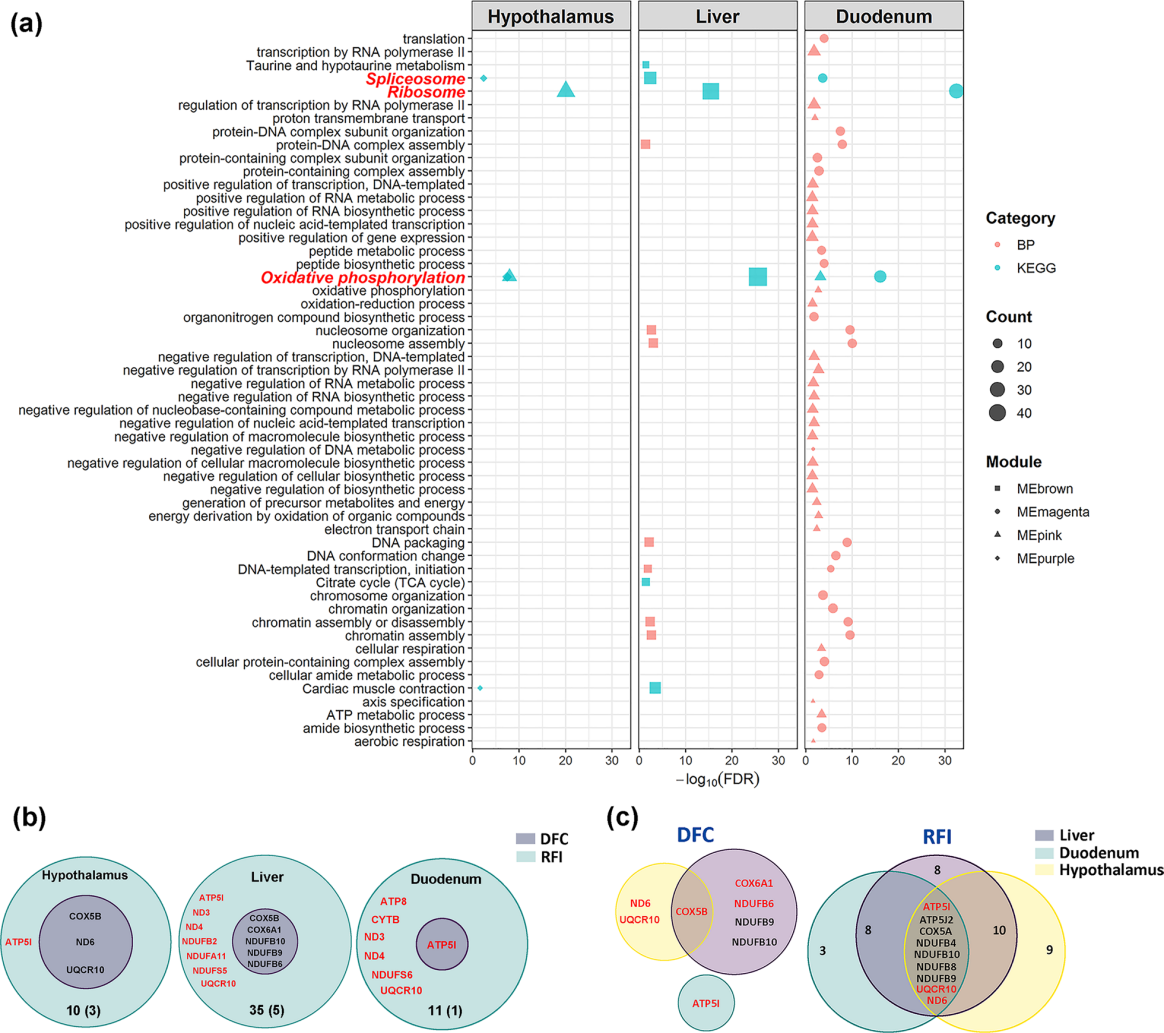
The hub genes in the oxidation phosphorylation pathway related to the negative heterosis for feed intake (DFC) and residual feed intake (RFI) in the hypothalamus, liver, and duodenum mucosa. **a** Significant biological processes and KEGG pathways enriched among genes harbored in the modules that are negatively related to feed intake and efficiency. Descriptions in red represent the KEGG pathway shared by the three tissues. **b** The Venn plot for the hub genes related to both DFC and RFI in each tissue. Genes in red are uniquely non-additively expressed genes in crossbred YW. The total number and shared number of hub genes are shown at the bottom of the circle. (**c**) The Venn plot for the hub genes in the three tissues for DFC and RFI, respectively. Genes in red are non-additively expressed genes in each tissue. The numbers denote the number of genes in each region

Enrichment analyses for the genes in the significant modules identified 263, 129, and 277 significantly enriched GO terms and KEGG pathways for hypothalamus, liver, and duodenum mucosa, respectively (see Additional file 11: Table S10). Interestingly, the genes present in the five negatively correlated modules were simultaneously enriched for the spliceosome, ribosome, and OXPHOS pathways (Fig. 6a). By focusing on the OXPHOS pathway, we extracted 11, 16, 49, 9, and 22 genes for the five modules (MEpurple and MEpink for hypothalamus, MEbrown for liver, and MEpink and MEmagenta for duodenum mucosa, respectively) (see Additional file 12: Table S11). In view of the similarity between the five modules that were negatively correlated with DFC and RFI (the OXPHOS-modules), we evaluated the preservation of each module among the three tissues by conducting permutation tests. By projecting the transcriptome of one tissue onto the network of the other tissues, we found that the OXPHOS-modules exhibited moderate preservation (2 < Zsummary < 10) for all three tissues (see Additional file 4: Figure S5). These results suggest that the transcriptional architecture that underlies the negative heterosis for feed intake and efficiency is conserved among the three tissues.

### Integrative analysis reveals key OXPHOS-regulators that are involved in heterosis for feed intake and efficiency

To further identify the key regulators in the molecular network underlying heterosis for DFC and RFI, we detected the hub genes and lncRNA that were highly connected to other gene/lncRNA and traits in the OXPHOS-modules using WGCNA. The genes or lncRNA were characterized by high gene significance (GS) defined as the correlation of gene expression profiles with an external trait and module membership (MM) values calculated by correlating its gene expression profile with the module eigengene of a given module. Then genes or lncRNAs with |GS|> 0.7 and |MM|> 0.7 within modules were regarded as hub genes. For DFC, we found 59 hub genes in the MEpurple module for hypothalamus, four hub genes in the MEpink module for hypothalamus, 42 hub genes in the MEbrown module for liver, one hub gene in the MEpink module for duodenum mucosa, and 36 hub genes in the MEmagenta module for duodenum mucosa (see Additional file 13: Table S12a). For RFI, we identified 119 hub genes in the MEpurple module for hypothalamus, 69 hub genes in the MEpink module for hypothalamus, 273 hub genes in the MEbrown module for liver, 39 hub genes in the MEpink module for duodenum mucosa and 95 hub genes in the MEmagenta module for duodenum mucosa (see Additional file 13: Table S12b). We then screened these hub genes against the OXPHOS pathway genes for each tissue and found for DFC and RFI, respectively, 3 and 10 overlapping genes in the hypothalamus, 5 and 35 overlapping genes in the liver, and 1 and 11 overlapping genes in the duodenum mucosa. Interestingly, DFC-related genes (*COX5B*, *ND6,* and *UQCR10* in the hypothalamus, *COX5B*, *COX6A1*, *NDUFB6*, *NDUFB9,* and *NDUFB10* in the liver, and *ATP5I* in the duodenum mucosa) were also correlated with RFI (Fig. 6b). Among these overlapping genes, *ATP5I* was a non-additive gene that was identified in the three tissues, which suggests that, in the duodenum mucosa, *ATP5I* could be a key regulator involved in the heterosis for DFC and RFI. Regarding the tissues, the non-additive gene *COX5B* was shared by both hypothalamus and liver and regulated DFC. For RFI, the shared genes between the three tissues included *ATP5I*, *ATP5J2*, *COX5A*, *ND6*, *NDUFB4*, *NDUFB8*, *NDUFB9*, *NDUFB10,* and *UQCR10*, with *ATP5I*, *ND6,* and *UQCR10* being dominant genes that regulate heterosis for RFI (Fig. 6c).

Based on the results of these integrative analyses, the ATP5I protein was selected for validation by Western blot in the liver tissue of WL, YW, and YY. The results show that the relative amount of ATP5I protein was low in WL and YW but high in YY (see Additional file 4: Figure S6), which is consistent with the expression levels observed for *ATP5I* in these tissues. Therefore, this key gene, which is involved in the OXPHOS pathway, could be a candidate target to regulate heterosis for feed intake and efficiency in laying chickens.

## Discussion

In this study, we constructed a reciprocal crossbred population between White Leghorn and Beijing You chicken and measured their daily feed intake and RFI from 43 to 46 weeks of age. Contrary to the findings reported by Bordas et al. [34], who reported similar heterosis values for RFI for two reciprocal crossbred populations from selected chicken lines with high and low RFI, we observed significantly different levels of heterosis between the two reciprocal crossbred populations for both feed intake and RFI. In animal production, whether heterosis for a trait is positive or negative depends on the practical significance of traits. We found that the YW crossbred consumed more feed and had a larger RFI than that of WY crossbreds, which are both unfavorable since they increase feed costs, and, thus, the YW crossbreds displayed negative heterosis for these traits. Thus, heterosis does not always result in hybrid vigor for a certain phenotype [35]. Compared to positive heterosis, which is widely used and studied, negative heterosis is usually neglected in breeding programs and genetic studies, although it frequently occurs in various agricultural and wild species [14].

Analysis of RNA-sequencing data is an effective tool for genomic studies in animal genetics and has been used to elucidate the molecular basis of heterosis in cattle [36], fish [37], and chicken [38]. However, most studies have focused on a single tissue or organ, which may not provide a comprehensive understanding of the interactive mechanisms that contribute to a phenotype and its heterosis [6]. For the first time, we provide a transcriptome atlas of three tissues that are associated with feed intake and efficiency (hypothalamus, liver, and duodenum mucosa) in hybrid chickens and their parents, allowing us to identify the shared and specific co-expression networks among these tissues underlying heterosis for feed intake and efficiency.

Our RNA-sequencing produced high proportions (overall mapping rate > 90%) of quality (Q20 > 98%) reads, which ensured the accuracy and reliability of the expression pattern analyses. The significantly larger number of expressed genes and lncRNAs found in the hypothalamus than in the liver and duodenum mucosa is similar to previous reports in humans and mouse [39], and indicates the central and sophisticated role that the hypothalamus plays in regulating body homeostasis [40]. As in the case of dominant or recessive alleles in classical genetics, non-additive genetic variance is due to nonlinear phenotypic effects of alleles at one locus [41]. Recent transcriptome studies have proposed dominance as the most promising molecular basis for heterosis in plants [42], animals [38], and model organisms [11]. Similarly, our analysis revealed that non-additivity is the major gene expression pattern across three tissues in YW, which show a negative heterosis for both feed intake and RFI. These results suggest that the magnitude of the heterotic phenotype is related to the proportion of genes with non-additive expression [14]. Although the global gene expression profile was similar among the three tissues, the non-additive genes that mostly differentially expressed between purebreds were expressed in a tissue-specific pattern in the crossbreds indicating that a majority of the differentially expressed genes between purebreds do not exhibit a consistently non-additive expression pattern in different tissues [43].

In the present study, the non-additively expressed genes found for the duodenum mucosa of WY were significantly enriched in the cellular component biogenesis as previously reported in dwarf laying hens with divergent RFI [44], suggesting that the cellular component of intestine mucosa is critical for modulating RFI. Compared to YW, we speculated that the positive heterosis for RFI observed for WY was less affected by feed intake and behavior that are regulated by the regulatory network of hypothalamus [45], since no GO term or KEGG pathway was enriched among non-additive genes in the hypothalamus. In contrast to WY, which showed negative heterosis for feed intake and efficiency, we found that mitochondrial components and oxidative phosphorylation pathways were significantly enriched among genes that were non-additively expressed for YW in the three tissues, which indicates the special and crucial roles of energy metabolism in the negative heterosis of feed intake and RFI. The two traits were highly correlated with each other genetically and phenotypically [16]. In the 1960s to 1970s, mitochondrial amalgamation and complementation were reported to be associated with heterosis in crops [46, 47] but the underlying mechanism was not identified due to the limits of the molecular approaches used. Recently, researchers have linked growth-related traits of *Arabidopsis thaliana* hybrids [48] and chicken crossbreds [14] with energy production via oxidative phosphorylation. Our findings imply that oxidative phosphorylation has a pleiotropic effect on the heterosis of the two traits.

To further validate the reliability and biological significance of the OXPHOS pathway in the negative heterosis of feed intake and efficiency for YW, we analyzed the mRNA-lncRNA expression data using the WGCNA method, which focused on associations between co-expression modules and investigated traits. Consistently, the OXPHOS pathway was identified in the co-expression modules that were significantly correlated with feed intake and RFI across the three tissues, indicating that the oxidative phosphorylation process plays an important role in regulating feed intake and RFI. Furthermore, the ribosome pathway that is shared by the three tissues was reported to be associated with feed efficiency and energy balance in dairy cattle [49–51], which supports the robustness of our profiling data and bioinformatics analysis. Co-expression of the ribosome pathway with the OXPHOS pathway is expected to participate in protein synthesis, an energy-demanding process in which one mole of a polypeptide bond during protein synthesis requires ~ four ATP [52]. Highly connected hub genes in a module significantly associated with traits play essential roles in biological pathways and have been suggested as potential indicators of feed efficiency [58]. In our study, the non-additively expressed hub genes, which play an important role in the proton channel of ATP synthase to facilitate electron flow through the respiratory chain and provide energy for ATP synthesis, have been reported to be involved in the regulation of feed intake and feed efficiency, including *UQCR10* [59], *NDUFB9* [60], *ATP5J2* [49], *COX5B* [61], *COX6A1* [62], and *ND6* [63].

The energy of a eukaryotic cell is mainly (~ 90%) generated through oxidative phosphorylation in the mitochondria [53]. We observed that the level of expression of the OXPHOS-related genes was higher in the parental breed with the lower feed intake and RFI and lower in the YW crossbred, which had negative heterosis for feed intake and RFI. These findings are consistent with previously reported studies for expression in the rumen epithelium of low RFI steers [54, 55] and on the breast muscle of low RFI chickens [56]. Thus, the decreased levels of expression of OXPHOS-related genes that we observed for *ATP5I* indicate that crossbred YW may have a lower protein expression of ATP5I than purebred YY, as validated by Western blot, and lower ATP production, which impairs metabolic capacity to capture energy and nutrients from feeds consumed, resulting in higher feed intake [57]. Therefore, we measured feed intake/efficiency and the expression level of OXPHOS-related genes in parental breed in advance and selected efficient breeds with highly expressed OXPHOS-related genes as maternal parent were expected to improve feed efficiency in the crossbreds. However, gene expression profiles in multiple tissues are dynamic and breed-specific [61]. Further experiments are needed to confirm if the contributions of non-additively expressed genes and their related oxidative phosphorylation persist over a longer laying period in different crossbreds of laying chickens.

## Conclusions

Heterosis for feed intake and efficiency differed between reciprocal chicken crosses, with WY showing positive heterosis for RFI, and YW showing negative heterosis for both feed intake and RFI. A comprehensive transcriptome atlas of mRNA-lncRNA was developed for tissues related to feed intake and efficiency, i.e. hypothalamus, liver, and duodenum mucosa. Genome-wide expression pattern analysis showed that non-additivity is the major mode of inheritance for the three tissues in YW crossbred chickens, which had negative heterosis for both feed intake and RFI. The non-additive lncRNA and genes that were down-regulated in these tissues for YW are involved in the biological process of oxidative phosphorylation that reduces ATP production, which contributes to the negative heterosis for feed intake and RFI observed for this cross. Our findings imply that measurements of OXPHOS-related gene expression in tissues that are associated with feed efficiency may be valuable for screening candidate parental breeds or lines, thereby facilitating a rational choice of suitable material for producing crossbred chickens.

### Supplementary Information

**Additional file 1: Table S1.** Sampling and sequencing summary. The data provide sample information and sequence statistics including clean reads, clean bases, Q20, Q30, GC content and overall mapping rate.

**Additional file 2: Table S2.** Expression patterns of differentially-expressed genes in the hypothalamus, liver and duodenum mucosa. The data provide the number of identified genes for different expression patterns including above parent, above high-parent, high-parent, low-parent, below low-parent, below parent and additive.

**Additional file 3: Table S3.** Summary of the multivariate linear model for the calculation of RFI

**Additional file 4: Figure S1.** Phenotype diagram of feed intake and efficiency-related traits for White Leghorn (WL), Beijing You chicken (YY) and their reciprocal crosses (WY and YW). The dashed red line represents the mid-parent value. RFI: residual feed intake, DFC: daily feed intake, FCR: feed conversion ratio and DEM: daily egg mass. ** indicate significant value P < 0.01. **Figure S2.** Principal component analysis of genes **a** and lncRNA **b** in the hypothalamus, liver and duodenum mucosa of White Leghorn (WL), Beijing You chicken (YY) and their reciprocal crosses (WY and YW). **Figure S3.** lncRNA inheritance in the hypothalamus, liver and duodenum mucosa for the reciprocal crosses (WY and YW). **a** Proportion of additive, dominant and over-dominant genes in three tissues. **b** Venn plot for non-additively expressed genes in the WY (blue circle) and YW (orange circle). From left to right, the plots represent the hypothalamus, liver and duodenum mucosa, respectively. **Figure S4.** Determination of soft thresholds (power/β) that provided optimal scale-free topology indices of co-expression networks in the hypothalamus (**a**), liver (**b**) and duodenum mucosa (**c**). **Figure S5.** Preservation of co-expression networks between the hypothalamus, liver and duodenum mucosa. **a**–**c** Pairwise module preservation analyses with hypothalamus, liver and duodenum mucosa as reference, respectively. Dashed red and blue lines represent the Zsummary thresholds for strong (Zsummary > 10) and weak to moderate (2 < Zsummary < 10) preservation levels, respectively. Colored dots represent the corresponding modules in the reference network, and the module size is the number of overlapping genes within each reference module. **Figure S6.** ATP5I protein relative quantity in the liver determined by Western blot in White Leghorn (WL), Beijing You chicken (YY) and crossbred YW.

**Additional file 5: Table S4.** Expression patterns of differentially-expressed lncRNA in the hypothalamus, liver and duodenum mucosa. The data provide the number of identified lncRNA for different expression patterns including above parent, above high-parent, high-parent, low-parent, below low-parent, below parent and additive.

**Additional file 6: Table S5.** Gene ontology (GO) term and KEGG pathway for non-additively expressed genes in the hypothalamus, liver and duodenum mucosa.

**Additional file 7: Table S6.** The protein coding genes (cis-genes) located within 100 kb of lncRNA.

**Additional file 8: Table S7.** Pearson correlations between protein-coding genes (trans-genes) and lncRNA with an absolute value $\ge$ 0.95

**Additional file 9: Table S8.** T he non-additively expressed lncRNA-gene pairs for the crossbred YW. The data provide non-additively expressed gene-lncRNA pairs identified in the hypothalamus, liver, and duodenum mucosa of YW. Both the gene and lncRNA of each pair were YW-specific non-additively expressed.

**Additional file 10: Table S9.** Non-additive genes and lncRNA in the hypothalamus, liver and duodenum mucosa for YW.

**Additional file 11: Table S10.** Significantly enriched GO terms and KEGG pathways for the trait-related modules genes in the hypothalamus (**a**), liver (**b**) and duodenum mucosa (**c**). The data provide GO terms and KEGG pathways with an adjusted *P* value lower than 0.05 for genes harbored in the WGCNA modules that are correlated with daily feed intake and residual feed intake.

**Additional file 12: Table S11.** Significant module genes that are enriched in the oxidation phosphorylation pathway in the hypothalamus, liver and duodenum mucosa. The data provided oxidation phosphorylation pathway enriched by genes in the WGCNA modules that significantly correlated with daily feed intake and residual feed intake.

**Additional file 13: Table S12.** WGCNA hub genes of the OXPHOS-modules for daily feed intake (**a**) and residual feed intake (**b**).

## Data Availability

The RNA sequencing data are available from the Sequence Read Archive (https://ngdc.cncb.ac.cn/) with BioProject number PRJCA012606.
