# Supplementary material for: The mRNA-lncRNA landscape of multiple tissues uncovers key regulators and molecular pathways that underlie heterosis for feed intake and efficiency in laying chickens
Source: Genet Sel Evol. 2023 Oct 6;55:69. doi: 10.1186/s12711-023-00834-x (PMC10559425; doi:10.1186/s12711-023-00834-x)
Supplement: Supplementary file 3 — Additional file 3: Table S3. Summary of the multivariate linear model for the calculation of RFI [file 12711_2023_834_MOESM3_ESM.docx]

**Table S3 The summary of multivariate linear model for the calculation of RFI**

| lm(formula = dat$DFC ~ dat$DEM + dat$MMBW + dat$BWG) |
| --- |
|  |
| Residuals: |
| Min 1Q Median 3Q Max  -46.866 -6.278 0.229 6.090 48.394 |
|  |
| Coefficients: |
| Estimate Std. Error t value Pr(>\|t\|)  (Intercept) 18.67194 3.87293 4.821 1.68e-06 ***  dat$DEM 0.61532 0.03309 18.595 < 2e-16 ***  dat$MMBW 0.18574 0.01277 14.544 < 2e-16 ***  dat$BWG 0.59588 0.10040 5.935 4.19e-09 *** |
| --- |
| Signif. codes: 0 ‘***’ 0.001 ‘**’ 0.01 ‘*’ 0.05 ‘.’ 0.1 ‘ ’ 1 |
|  |
| Residual standard error: 10.83 on 900 degrees of freedom |
| Multiple R-squared: 0.3817, Adjusted R-squared: 0.3796 |
| F-statistic: 185.2 on 3 and 900 DF, P-value: < 2.2e-16 |
