# Supplementary material for: The mRNA-lncRNA landscape of multiple tissues uncovers key regulators and molecular pathways that underlie heterosis for feed intake and efficiency in laying chickens
Source: Genet Sel Evol. 2023 Oct 6;55:69. doi: 10.1186/s12711-023-00834-x (PMC10559425; doi:10.1186/s12711-023-00834-x)

Figure S1. Phenotype diagram of feed intake and efficiency related traits for White Leghorn (WW), Beijing You chicken (YY) and their reciprocal crosses (WY and YW). The dashed red line represents the mid-parent value. RFI, DFC, FCR and DEM represent residual feed intake, daily feed intake, feed conversion ratio and daily egg mass, respectively. ** indicate highly significant (P < 0.01).

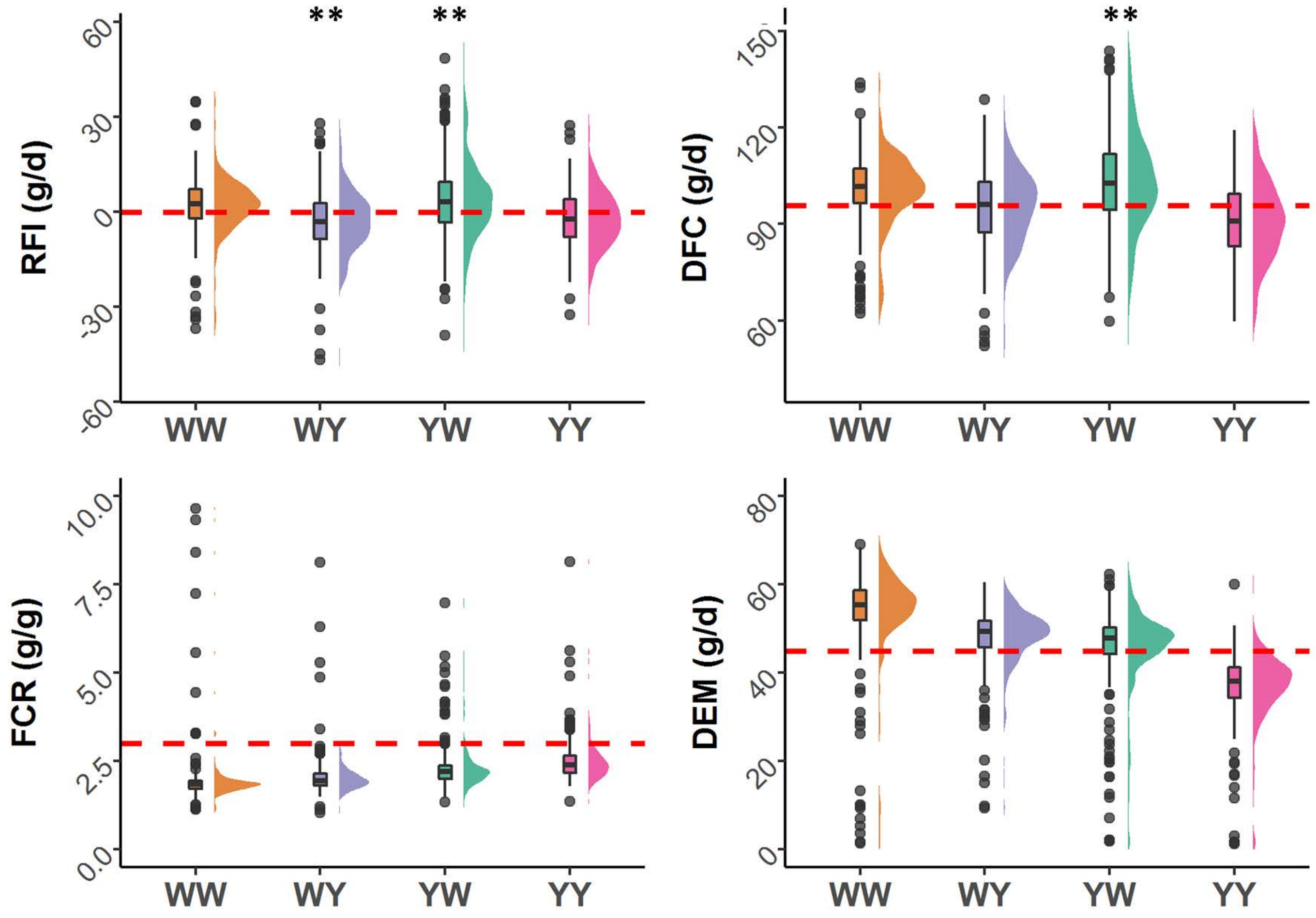

Figure S2. Principal component analysis of genes (a) and lncRNAs (b) in the hypothalamus, liver and duodenum mucosa for White Leghorn (WW), Beijing You chicken (YY) and their reciprocal crosses (WY and YW).

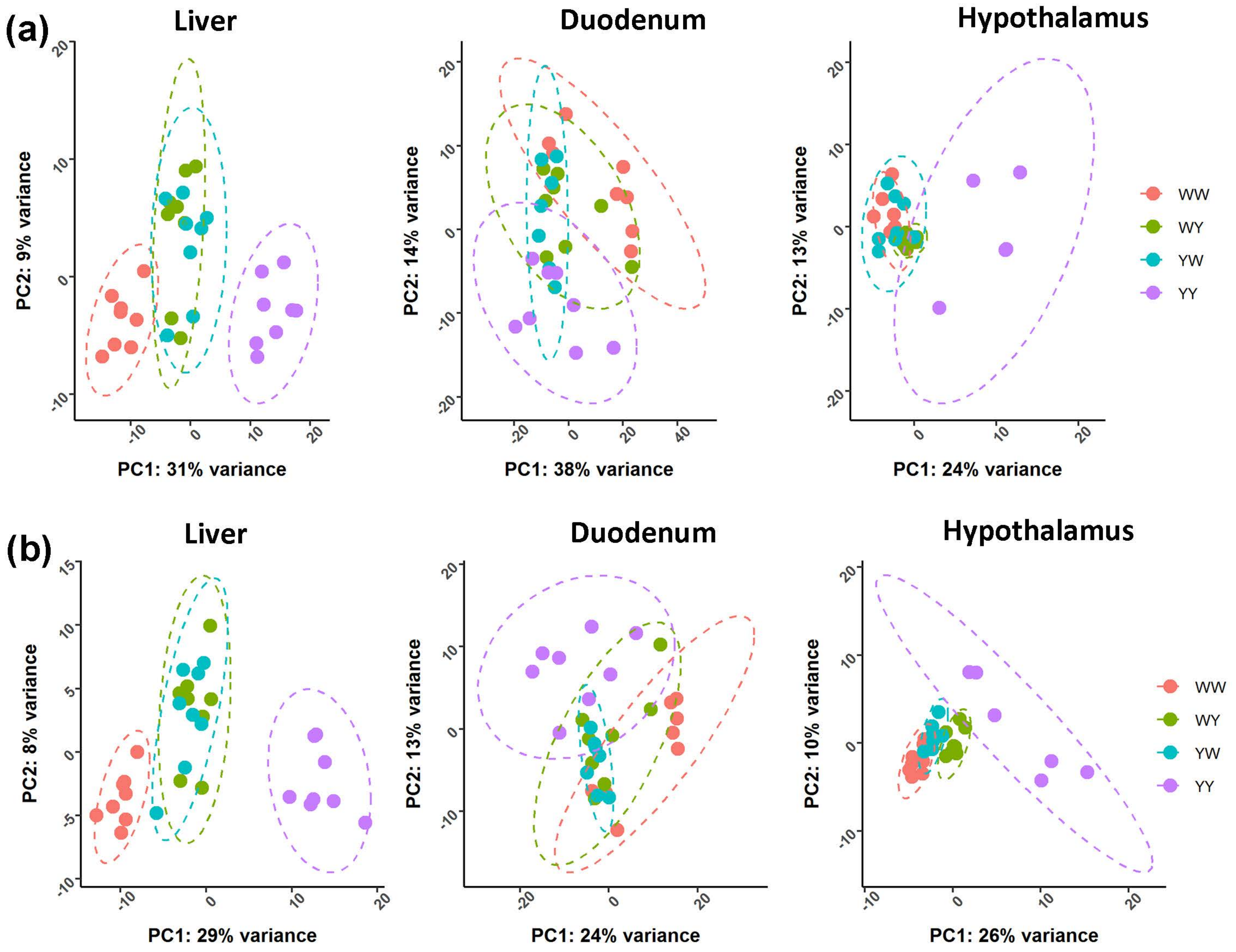

Figure S3. LncRNA inheritance of the hypothalamus, liver and duodenum mucosa in the reciprocal crosses (WY and YW). (a) The proportion of additive, dominant and over-dominant genes in three tissues. (b) The venn plot for nonadditive genes in the WY (blue circle) and YW (orange circle). From left to right, the plot represents the hypothalamus, liver and duodenum mucosa, respectively.

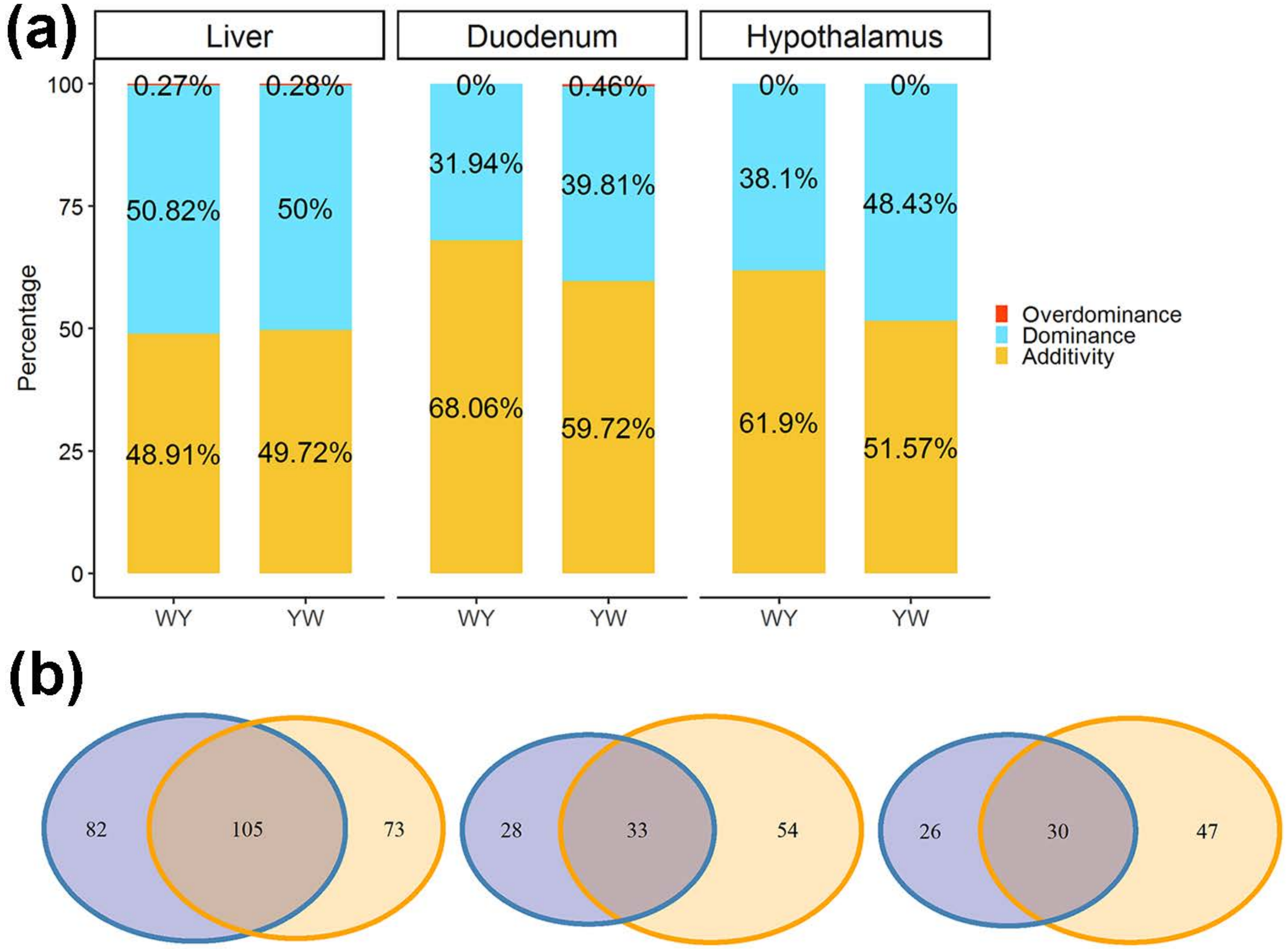

Figure S4. Determination of soft thresholds (power/β) that provided optimal scale-free topology indices of co-expression networks in the hypothalamus (a), liver (b) and duodenum mucosa. (c).

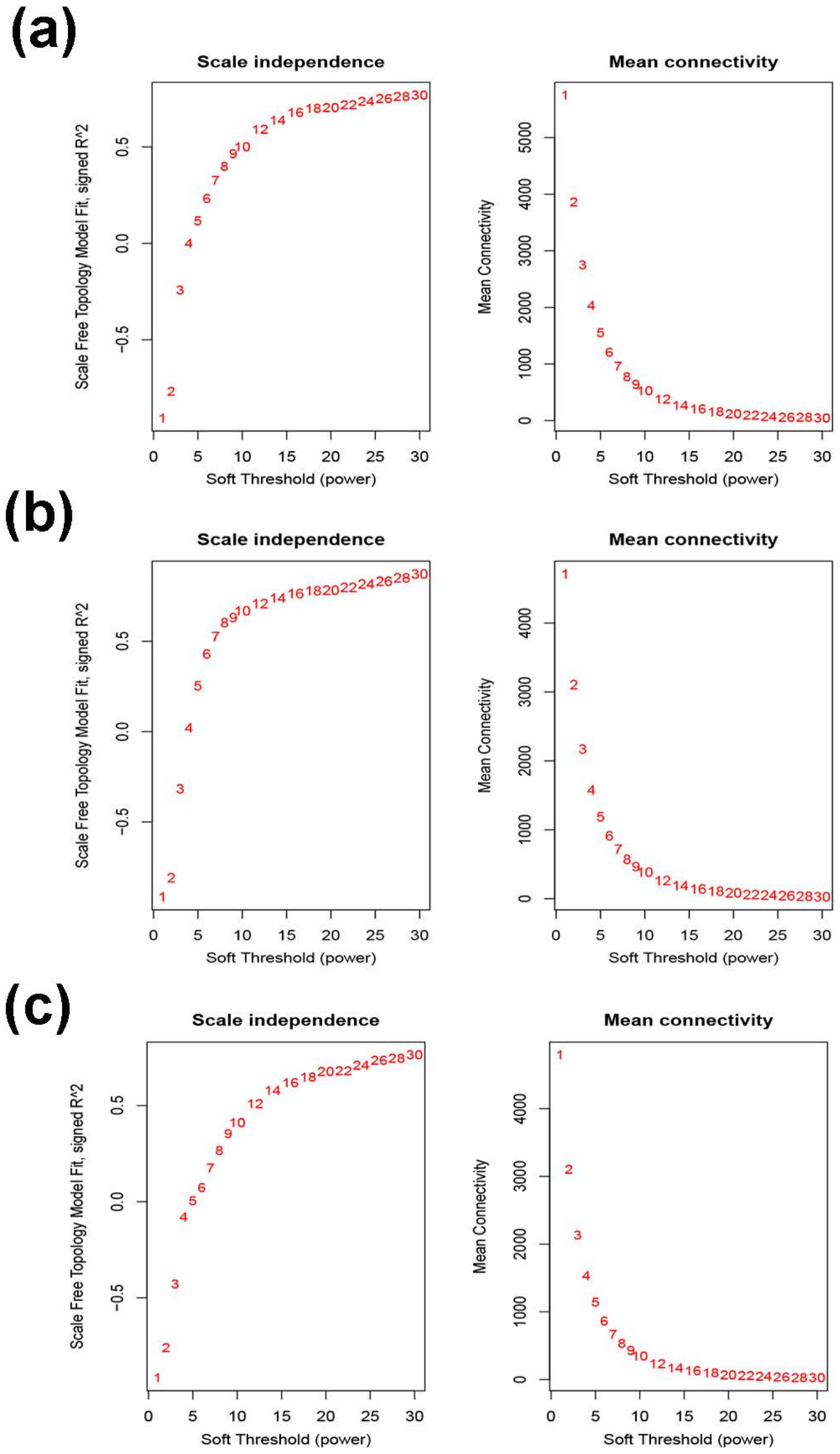

Figure S5. Preservation of co-expression networks among the hypothalamus, liver and duodenum mucosa. (a-c) Pairwised module preservation analyses with hypothalamus, liver and duodenum mucosa as reference, respectively. Dashed red and blue lines represent the Zsummary thresholds for strong (Zsummary > 10) and weak to moderate (2 < Zsummary < 10) preservation levels, respectively. Colored dots represent the corresponding modules in the reference network, and the module size is the number of overlapped genes within each reference module.

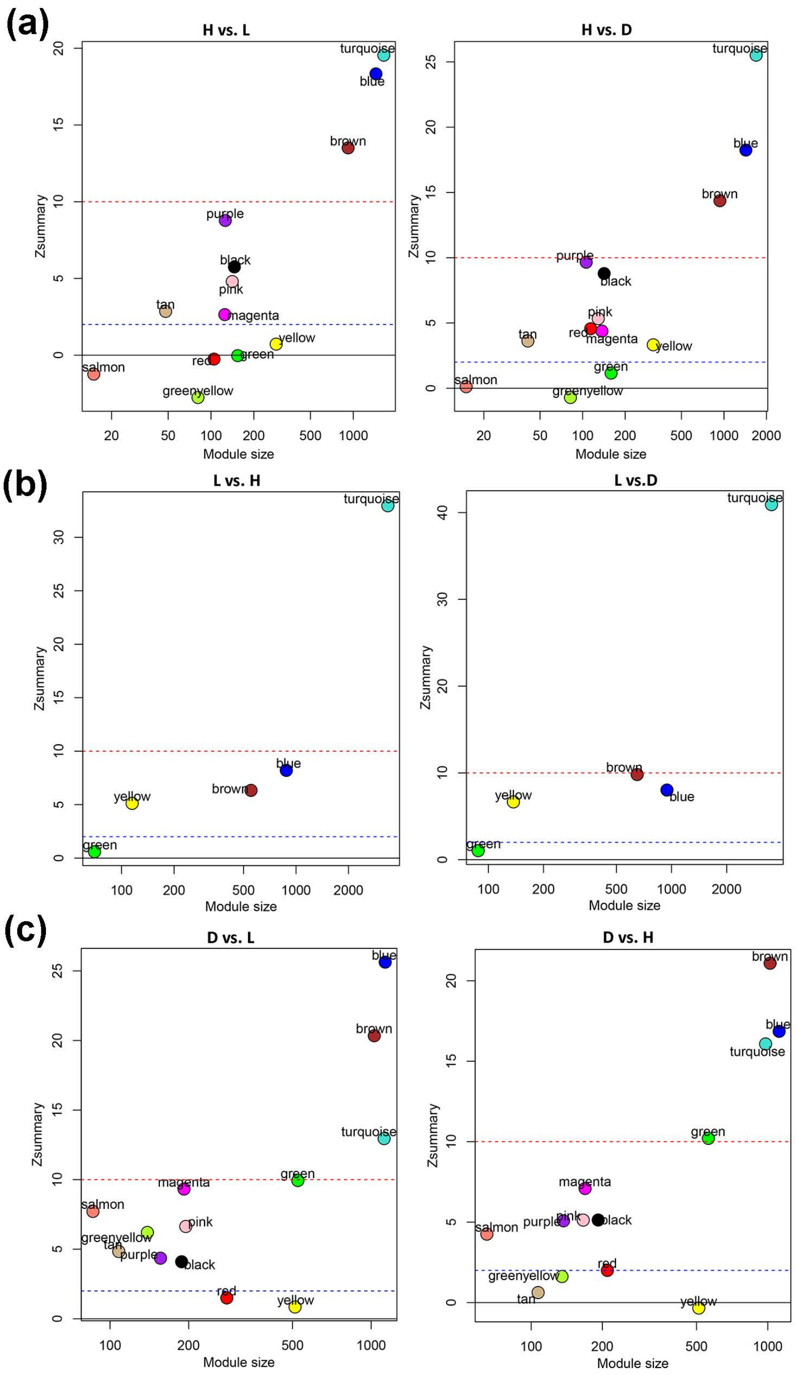

Figure S6. The protein expression of *ATP5I* in the liver determined by Western blotting for White Leghorn (WW), Beijing You chicken (YY) and crossbred YW.

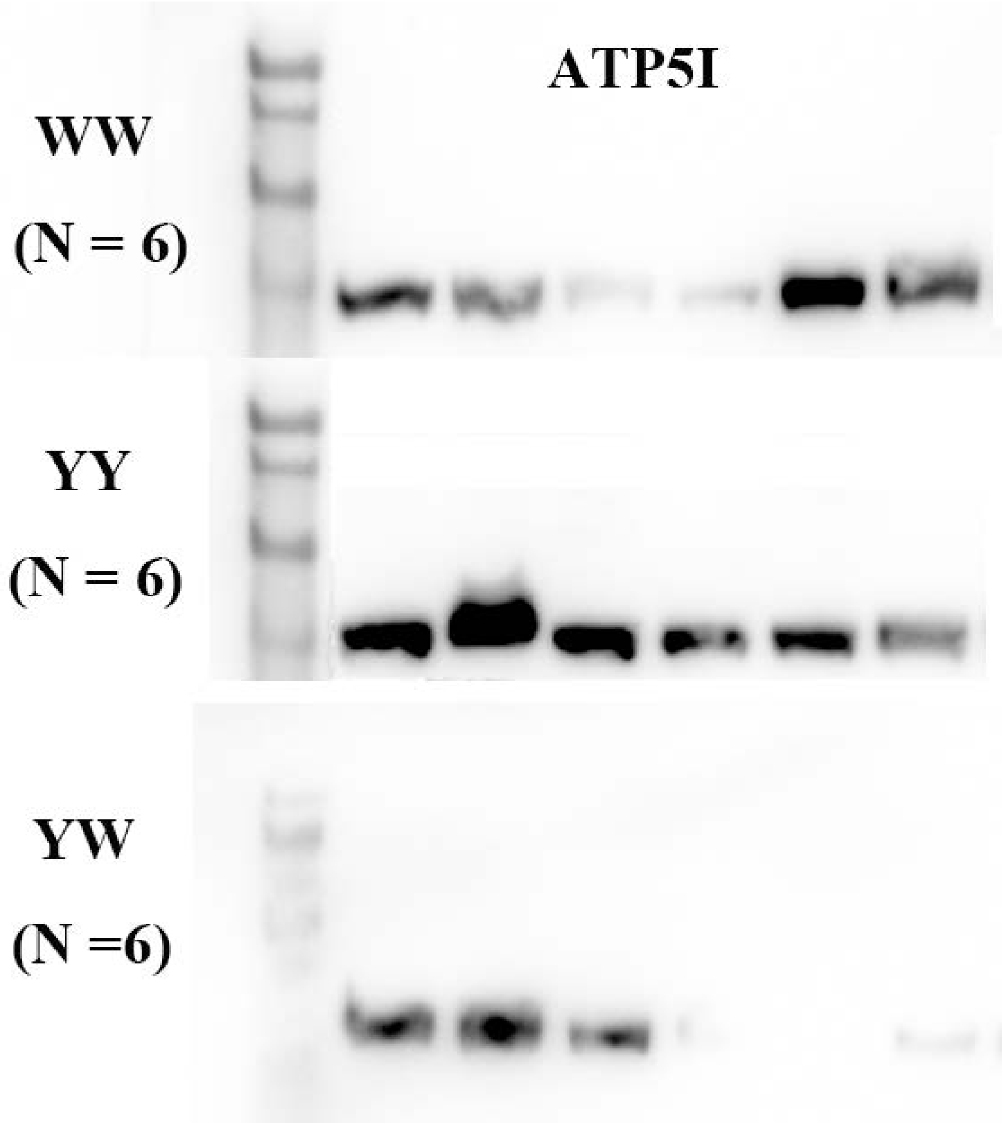

Supplement: Supplementary file 4 — Additional file 4: Figure S1. Phenotype diagram of feed intake and efficiency-related traits for White Leghorn (WL), Beijing You chicken (YY) and their reciprocal crosses (WY and YW). The dashed red line represents the mid-parent value. RFI: residual feed intake, DFC: daily feed intake, FCR: feed conversion ratio and DEM: daily egg mass. ** indicate significant value P < 0.01. Figure S2. Principal component analysis of genes a and lncRNA b in the hypothalamus, liver and duodenum mucosa of White Leghorn (WL), Beijing You chicken (YY) and their reciprocal crosses (WY and YW). Figure S3. lncRNA inheritance in the hypothalamus, liver and duodenum mucosa for the reciprocal crosses (WY and YW). a Proportion of additive, dominant and over-dominant genes in three tissues. b Venn plot for non-additively expressed genes in the WY (blue circle) and YW (orange circle). From left to right, the plots represent the hypothalamus, liver and duodenum mucosa, respectively. Figure S4. Determination of soft thresholds (power/β) that provided optimal scale-free topology indices of co-expression networks in the hypothalamus (a), liver (b) and duodenum mucosa (c). Figure S5. Preservation of co-expression networks between the hypothalamus, liver and duodenum mucosa. a–c Pairwise module preservation analyses with hypothalamus, liver and duodenum mucosa as reference, respectively. Dashed red and blue lines represent the Zsummary thresholds for strong (Zsummary > 10) and weak to moderate (2 < Zsummary < 10) preservation levels, respectively. Colored dots represent the corresponding modules in the reference network, and the module size is the number of overlapping genes within each reference module. Figure S6. ATP5I protein relative quantity in the liver determined by Western blot in White Leghorn (WL), Beijing You chicken (YY) and crossbred YW. [file 12711_2023_834_MOESM4_ESM.pdf]
